# Modeling the two-locus architecture of divergent pollinator adaptation: how variation in *SAD* paralogs affects fitness and evolutionary divergence in sexually deceptive orchids

**DOI:** 10.1002/ece3.1378

**Published:** 2015-01-04

**Authors:** Shuqing Xu, Philipp M Schlüter

**Affiliations:** 1 Department of Molecular Ecology, Max Planck Institute for Chemical EcologyHans-Knöll-Straße 8, D-07745, Jena, Germany; 2 Institute of Systematic Botany, University of ZurichZollikerstrasse 107, CH-8008, Zürich, Switzerland

**Keywords:** Divergent selection, ecological speciation, molecular basis of adaptation, plant fitness, plant–pollinator interaction, sexually deceptive orchids, speciation genes, stearoyl–ACP desaturases

## Abstract

Divergent selection by pollinators can bring about strong reproductive isolation via changes at few genes of large effect. This has recently been demonstrated in sexually deceptive orchids, where studies (1) quantified the strength of reproductive isolation in the field; (2) identified genes that appear to be causal for reproductive isolation; and (3) demonstrated selection by analysis of natural variation in gene sequence and expression. In a group of closely related *Ophrys* orchids, specific floral scent components, namely *n*-alkenes, are the key floral traits that control specific pollinator attraction by chemical mimicry of insect sex pheromones. The genetic basis of species-specific differences in alkene production mainly lies in two biosynthetic genes encoding stearoyl–acyl carrier protein desaturases (SAD) that are associated with floral scent variation and reproductive isolation between closely related species, and evolve under pollinator-mediated selection. However, the implications of this genetic architecture of key floral traits on the evolutionary processes of pollinator adaptation and speciation in this plant group remain unclear. Here, we expand on these recent findings to model scenarios of adaptive evolutionary change at *SAD2* and *SAD5*, their effects on plant fitness (i.e., offspring number), and the dynamics of speciation. Our model suggests that the two-locus architecture of reproductive isolation allows for rapid sympatric speciation by pollinator shift; however, the likelihood of such pollinator-mediated speciation is asymmetric between the two orchid species *O. sphegodes* and *O. exaltata* due to different fitness effects of their predominant *SAD2* and *SAD5* alleles. Our study not only provides insight into pollinator adaptation and speciation mechanisms of sexually deceptive orchids but also demonstrates the power of applying a modeling approach to the study of pollinator-driven ecological speciation.

## Introduction

Linking specific genes to fitness differences is one of the main objectives of evolutionary biology, with recent research programs increasingly aiming at uncovering the genes that underlie adaptation and ecological speciation (Lexer and Widmer 2008; Presgraves 2010; Nosil and Schluter 2011; Ostevik et al. 2012). It is important to understand the genetic architecture of phenotypic traits, because it may influence their evolutionary trajectories by facilitating or constraining the way in which traits can be altered by selection. Knowledge of the genetic basis of adaptive traits allows us to infer the accessibility of different evolutionary paths, and the genetic changes and selective pressures that are necessary to effect evolutionary change. Although potentially powerful to address questions that are not directly amenable to experimentation, field-derived fitness estimates of specific alleles underlying phenotypic traits have so far only rarely been used for modeling the trajectories and possible outcomes of evolutionary change (but see Hopkins and Rausher 2014).

Significant progress has recently made in uncovering the genetic basis of adaptation to pollinators that likely enables ecological speciation in a specialized pollination system, namely sexually deceptive orchids (Xu et al. 2012b). Unlike most animal-pollinated angiosperms, flowers of these orchids do not provide a reward to their pollinators; they are therefore unattractive to typical foraging flower visitors. Rather, these plants are adapted to use the species-specific sexual signals of female insects to attract male insects, which subsequently attempt to copulate with flowers and thereby remove or deliver the orchids' pollen packets (pollinia) (Xu et al. 2012b). The high species specificity of pollinator interaction in this genus has allowed the identification of the chemical signals that trigger pollinator behavior and thus underlie the plant's adaptation to its pollinator (Schiestl et al. 2000; Mant et al. 2005b). Specifically, in many species of the Mediterranean orchid genus *Ophrys*, the mimicked sexual signal is made up of cuticular hydrocarbons, most importantly alkenes, that constitute the pollinator females' sex pheromones (Ayasse et al. 2011).

A recent study tracking pollen movement in the field has demonstrated that reproductive isolation is indeed mediated by floral signals (floral isolation), mostly due to hydrocarbon profile differences that are associated with differences in specific pollinator attraction among the closely related species *O. sphegodes* miller,*O. exaltata* subsp. *archipelagi* (gölz & H. R. reinhard) del prete, and *O. garganica* nelson ex O. & E. danesch (Xu et al. 2011). All of these species cooccur in mosaic sympatry (sensu Mallet et al. 2009; Xu et al. 2012b, 2011) and overlap in their flowering times; they are diploids which are interfertile and produce putatively viable seeds, implying that postmating barriers – as far as it was possible to test them – do not play a prominent role in reproductive isolation. In contrast, the floral hydrocarbon mixtures used by the different species differ significantly, and floral isolation has therefore been implicated as the most important premating barrier (Xu et al. 2011; Sedeek et al. in press[Bibr b20]). In particular, *O. sphegodes* and *O. exaltata* differ in their production of alkenes with different double-bond positions: *O. sphegodes* produces mostly 9- and 12-alkenes, whereas *O. exaltata* produces high levels of 7-alkenes. Floral odor extracts by themselves are attractive to pollinators (Schiestl et al. 2000; Mant et al. 2005b; Vereecken and Schiestl 2008), and the solitary bee *Andrena nigroaenea* (kirby 1802) – the pollinator of *O. sphegodes* – appears to be attracted to 9- and 12-alkenes, whereas 7-alkenes reduce this attraction (Xu et al. 2012a). Conversely, *Colletes cunicularius* (linnaeus 1761), the pollinator of *O. exaltata*, is attracted to 7-alkenes, whereas addition of 9- and 12-alkenes to the odor blend reduces its attractiveness (Mant et al. 2005b; Xu et al. 2012a). This is expected to result in pollinator-imposed divergent selection for different odor bouquets between these two orchid species (Xu et al. 2012a).

Two desaturase genes, *SAD2* and *SAD5*, have recently been found to be responsible for controlling 9-/12-alkene and 7-alkene production, respectively (Schlüter et al. 2011b; Xu et al. 2012a). The expression and enzymatic activity of SAD2 are typically high in *O. sphegodes* and low in *O. exaltata*, because of different predominant alleles in the two species that are associated with (1) *cis*-linked gene expression; and (2) protein functional differences (Schlüter et al. 2011b; Xu et al. 2012a). In contrast, the expression of *SAD5* is high in *O. exaltata* and low in *O. sphegodes*. In addition, *SAD5* expression may further be modified a the *trans*-acting dominant suppressor (Xu et al. 2012a); although both genes appear to have a major effect on pollinator attraction by orchid flowers (Schlüter et al. 2011b; Xu et al. 2012a), their relative effects on pollination might not be the same. Furthermore, it is currently unknown how the genetic architecture of alkene composition influences the possible outcomes of the evolutionary processes of pollinator adaptation and speciation. Here, by modeling the fitness effects (i.e., number of offspring) and evolution of these two adaptive loci, we ask how the predicted contributions of *SAD2* and *SAD5* to pollination compare, and which possible routes for evolutionary change in plant–pollinator associations may be plausible in *Ophrys* orchids given the opposing effect of these two loci on divergent pollinator adaptation. Furthermore, we evaluate the effect of a potential genetic modifier of desaturase expression on the possible evolutionary outcomes of pollinator-driven divergent adaptation.

## Materials and Methods

### Rationale for modeling: two-locus architecture and biosynthesis of alkene double-bonds in the study system

Alkene double-bond position discriminates the pollinator-attractive odors of *O. sphegodes* and *O. exaltata*, the biochemical basis of which has recently been elucidated. Alkenes are thought to be produced via elongation of unsaturated fatty acid (FA) precursors, and the introduction of a double-bond into a saturated FA intermediate – termed desaturation – is the crucial enzymatic step that determines the double-bond in the alkene (Schlüter and Schiestl 2008; Perera et al. 2010; Schlüter et al. 2011b; Haslam and Kunst 2013). This reaction is catalyzed by a soluble, nuclear-encoded, and plastid-localized protein, stearoyl–acyl carrier protein desaturase (SAD) that acts on an acyl group (like the common C_18_ saturated FA, stearate) linked to acyl carrier protein (ACP) (Shanklin and Cahoon 1998; Schlüter et al. 2011b). Six *SAD* gene family members (*SAD1* – *SAD6*) have been identified from *Ophrys* orchids (Schlüter et al. 2011b; Xu et al. 2012a; Sedeek et al. 2013), and these desaturases comprise three lineages: the *SAD1*/*2*,*SAD3,* and *SAD4*/*5*/*6* clades (Xu et al. 2012a). Among these, *Ophrys SAD3* most likely represents a ubiquitously expressed housekeeping gene without direct involvement in alkene biosynthesis (Schlüter et al. 2011b; Xu et al. 2012a). By contrast, *O. sphegodes SAD2* is a florally expressed gene that is statistically associated with the production of 9- and 12-alkenes during flower development (Schlüter et al. 2011b). It can catalyze the desaturation of C_18_ and C_16_ FA-ACP in vitro, the elongation of which would result in the production of 9- and 12-alkenes, respectively (Schlüter et al. 2011b). Conversely, the gene *SAD5* is highly expressed in *O. exaltata* flowers and is strongly associated with 7-alkenes in natural orchid populations (Xu et al. 2012a); the expression of this gene, however, is suppressed in interspecific F_1_ hybrids, suggesting the presence of a *trans*-acting dominant suppressor of *SAD5* expression in *O. sphegodes*, henceforth called *SUS*. The patterns of differentiation in terms of sequence and/or gene expression at the *SAD2* and *SAD5* loci (which are absent for genes not associated with floral alkene biosynthesis; cf. Sedeek et al. in press) suggest that they experience divergent selection between species (Xu et al. 2012a). In reality, several alleles for each of the six *SAD* paralogs exist in nature (Xu et al. 2012a). In particular, in some (but not all) orchid populations, *SAD1* alleles may contribute to 9-/12-alkene biosynthesis (which is predominantly explained by SAD2 activity), and *SAD6* may sometimes contribute to the production of 7-alkenes (which is strongly associated with *SAD5*) (Xu et al. 2012a). However, for simplicity, we will ignore these minor contributors here and focus only on *SAD2* and *SAD5*, the genes with the largest overall effect on alkene biosynthesis, because they are expected to be primarily responsible for the divergent adaptations to different pollinators between the two orchid species.

### Modeling approach

**Figure 1:**
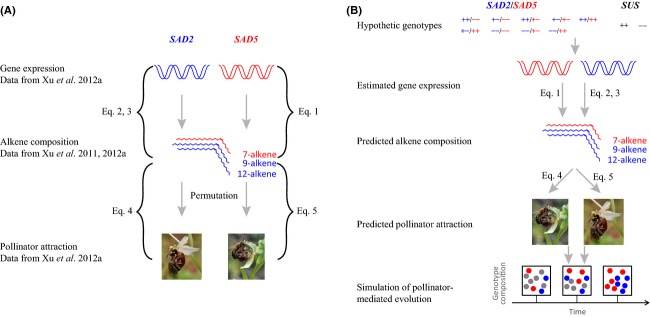
Schematic overview of data and approach used for modeling pollinator attraction. (A) Data and publications that were used for deriving the equations that represent the correlations between gene expression of *SAD2/SAD5* and alkene composition, as well as between alkene composition and pollinator attraction. Equations 1-5 refer to the equations given in the text. To derive Eqs. 4 and 5, floral scent and pollinator attraction data from a previous study (Xu et al. 2012a) were permutated 300 times. (B) Schematic diagram of the model that was built to predict pollinator attraction given different hypothetical genotypes of *SAD2* and *SAD5*.

Here, we model alkene composition of the two (diploid; Xu et al. 2011) orchid species as a function of the alleles of *SAD2* and *SAD5*, using previously published data (Schlüter et al. 2011b; Xu et al. 2011, 2012a). An overview of our modeling approach and the data used for building the model are illustrated in Figure1. Briefly, we (1) model the observed relationship between *SAD* allelic expression and odor (alkene) data; (2) link odor data to pollinator attractiveness, hence linking *SAD* gene expression and pollination; (3) predict pollinator attraction for all possible *SAD2*/*SAD5* genotypes; and (4) simulate pollination success and population genetic composition of *SAD* genotypes through time. As there is no indication of postmating reproductive barriers in the study system (Xu et al. 2011; Sedeek et al. in press), pollination success is taken to be an estimate of overall plant fitness because it is the primary factor assumed to affect the number of offspring any plant would produce. Here, *SAD2* is modeled to pleiotropically control 9- and 12-alkene levels which are high in *O. sphegodes*, and *SAD5* is modeled to control 7-alkene levels in *O. exaltata* (Xu et al. 2012a). In addition, *SAD5* expression was further modeled to be modified by the *trans*-acting dominant suppressor *SUS* (see Xu et al. 2012a). We assume that *SAD2*,*SAD5,* and *SUS* loci are nuclear genes that are inherited independently. Based on the findings of Xu et al. (2012a), flower attractiveness to the pollinator *A. nigroaenea* is expected to be a function of 9-/12-alkenes and 7-alkenes, which increase and decrease the likelihood of pollination, respectively. Flower attractiveness to the alternative pollinator *C. cunicularius* is modeled similarly, but with the opposite effect of the respective alkene double-bond classes. All data analyses were performed in R 3.0.1 (R Development Core Team 2013); input data, scripts for data analysis and simulations are available under doi:10.5061/dryad.sp58m from the Dryad digital repository (http://datadryad.org/).

#### Model step 1: gene expression to alkenes

Floral scent, gene expression, and pollinator behavior data were obtained from a previous study (Xu et al. 2012a; see Fig.1). To increase statistical power, we also included floral scent data from Xu et al. (2011). In step 1, we calculated the relationship between absolute amount of floral scent and gene expression using linear regression. The floral scent data were natural logarithm (*ln*) transformed, and gene expression data were square-root transformed. The StepAIC method (Venables and Ripley 2002) was used to select the best model.

#### Model step 2: linking odor data to pollinator attractiveness

In step 2, we fitted a generalized linear model (GLM) on pollinator attraction based on floral scent. To do so, observed pollinator attraction data from the field and floral scent of different individuals were permuted so as to incorporate the variance. In our previous study, four groups of floral scent were used to test pollinator attraction: *O. sphegodes* scent; *O. sphegodes* scent with addition of 7-alkenes; *O. exaltata* scent; and *O. exaltata* with addition 9- and 12-alkenes (Xu et al. 2012a). Within each group, we permuted the relationship between floral scent and pollination attraction 300 times to include the variance of observed pollinator attraction and floral scent data. The permuted data points were then used for calculating the relationship between floral scent and pollinator attraction using a GLM with Poisson distribution (link = “*log*”). Here, pollinator attraction was modeled as response variable of floral scent, and both linear and quadratic effects of floral scent on pollinator attraction were considered in the model selection process.

#### Model step 3: predicting pollinator attraction from the possible genotypes

**Figure 2:**
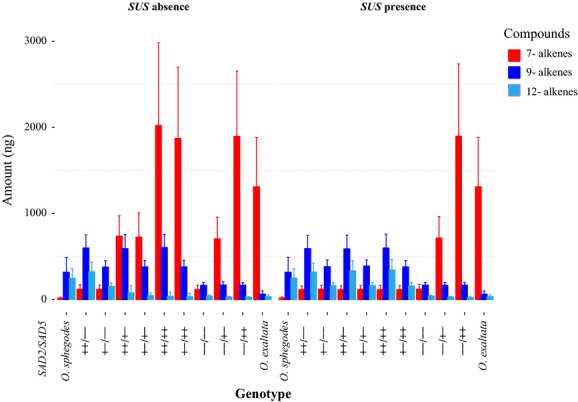
Predicted alkene composition given the indicated genotypes at *SAD2* and *SAD5* loci, with and without the presence of an additional *SUS* suppressor locus. Additionally, data from actual (so labeled) *O. sphegodes* and *O. exaltata* populations are shown for comparison. The *y*-axis represents the absolute amount of alkenes. Red, blue, and light blue colors refer to the sum of all 7-, 9-, and 12-alkenes, respectively. Each bar shows the mean (± standard deviation). Genotypes are listed as *SAD2* and *SAD5*, separated by a forward slash, where “+” refers to a functional allele (i.e., *SAD2A* or *SAD5A*), and “−” refers to a nonfunctional (or nonexpressed) allele.

In step 3, we predicted pollinator attraction for all possible *SAD* genotypes in the presence or absence of a fixed *SUS* locus that dominantly suppresses the expression of *SAD5*. To do so, two alleles were modeled for each investigated *SAD* locus based on previous allele group designations (Xu et al. 2012a): (1) a functional and expressed allele (denoted “+”, i.e., *SAD2A* or *SAD5A* alleles; allele group designation as in Xu et al. 2012a); and (2) a nonfunctional and/or nonexpressed allele (denoted “−”). We then evaluated all possible diploid genetic combinations (nine genotypes) at the two loci, *SAD2* and *SAD5*, and calculated the allelic expression values and expected alkene composition (Fig.2). This was based on the calculated correlation between *SAD2*/*SAD5* and alkene composition as well as the correlation between alkene composition and pollinator attraction. Pollinator attraction was scaled to fit the range 0–1, where 0 refers to the lowest and 1 to the highest pollinator attraction value observed in the field (Xu et al. 2012a). Field observations show that pollinator visitation rates of *O. exaltata* and *O. sphegodes* are similar (Xu et al. 2011). To achieve a robust estimation, we permuted the pollinator attraction calculation 500 times, each time allowing the estimated parameters (the effect of *SAD* expression on alkene composition and the effect of floral scent on pollinator attraction) in the model to vary within their 95% confidence intervals. Furthermore, in the *O. sphegodes* background, the evolutionary implication of the hypothetical *SUS* locus was investigated. This was carried out by running our predictions with and without the presence of *SUS* fixed in *O. sphegodes*, reflecting the two most extreme conditions possible for this genetic modifier.

#### Model step 4: population model simulated through time

Fourthly, we built a population model to evaluate the impact of *SAD2* and *SAD5* alleles as well as the *SUS* locus on reproductive isolation and the hypothetical speciation process between *O. sphegodes* and *O. exaltata*. The basic model was built under the following assumptions: an initial population size of 1000 individuals, setting the maximum population size to 1000 (i.e., if more than 1000 offspring were produced, only 1000 were kept); for each generation, a loss-of-function mutation rate per gene at 10^−5^ per generation per individual was used (to change “+” into “−” alleles), based on estimations from *Arabidopsis* (Ossowski et al. 2010); pollinator attraction for each individual depends on its floral alkene composition; total number of pollinators is 500 (including both *A. nigroaenea* and *C. cunicularius*); for each generation, each pollinator visits two individuals, leading to one pollination event, based on their pollinator attraction. Each pollination event produces 64 (4^3^; 3 loci with 2^2^ = 4 possible genotypes per diallelic locus) offspring that carry all possible genetic combinations of three genetic loci.

Based on this basic model, we varied pollinator composition (*A. nigroaenea* : *C. cunicularius*) as follows: 1:0; 3:1; 1:1; 1:3, and 0:1. We also simulated the following initial populations: F_1_ hybrids of *O. sphegodes* and *O. exaltata* (genotype +−/+− at *SAD2*/*SAD5*); a 1:1 mix of *O. sphegodes* and *O. exaltata*; an *O. sphegodes*-like (++/−−) population with a single *SAD5A* (“+”) allele present (in heterozygous state); an *O. exaltata*-like (−−/++) population with a single *SAD2A* (“+”) allele present (in heterozygous state). Hence, in the latter two cases, the model effectively begins at the time when a novel functional *SAD* allele has just arisen (i.e., de novo mutation). For each scenario, we simulated the population for 1000 generations and recorded genotype frequency every 20 generations. The model was also tested with different initial population sizes and a different number of pollinators, but no qualitative differences in results were found.

## Results

### Predicted alkene composition based on desaturase expression

Our previous data showed that expression of a functional *SAD2* allele (“+”; *SAD2* allele group A) is associated with the abundance of 9- and 12-alkenes, whereas expression of a functional *SAD5* allele (“+”; *SAD5* allele group A) is associated with the abundance of 7-alkenes (Xu et al. 2012a). Therefore, we first modeled the relationship between *SAD2*/*SAD5* allelic expression and 7-, 9-, and 12-alkene composition using a linear model based on data from Xu et al. (2012a). The amount of alkenes (denoted *Y*) was modeled as a function of the expression level of functional *SAD* alleles (denoted *X*; for details see Methods section). The amount of 7-alkenes only depended on *SAD5A* allele expression: 

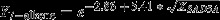
1

This formula could explain 52.2% of total variance in 7-alkenes. The amount of 9-alkenes was only significantly correlated with *SAD2A* allele expression: 

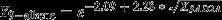
2

This formula could explain 40.12% of total variance in 9-alkenes. Interestingly, we found the amount of 12-alkenes to depend on the expression of both *SAD2A* and the interaction between *SAD2A* and *SAD5A*: 

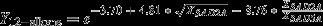
3

This formula could explain 70.34% of variance in 12-alkenes. It is noteworthy that the interaction term might potentially be explained by a metabolic interaction of SAD2 and SAD5 enzymes, because they might utilize the same substrates for the synthesis of 7- and 12-alkenes. Specifically, SAD2A synthesizes the precursor of 12-alkenes from a C_16_ FA-ACP substrate (Schlüter et al. 2011b), and it is conceivable (but so far untested) that SAD5A might also use an C_16_-ACP precursor for 7-alkene biosynthesis (Xu et al. 2012a).

### Alkene bouquet composition as a function of *SAD2*/*SAD5* genotype

The alkene composition for each genotype was predicted based on equations 3 and is shown is Figure2. *Ophrys exaltata* populations would be most closely represented by the genotype −−/++ (denoting two *SAD2* “−”-alleles and two “+”-alleles at the *SAD5* locus), whereas *O. sphegodes* would be most closely represented by the genotype ++/−−. The predicted alkene compositions of *O. sphegodes*-like (++/−−) and *O. exaltata*-like (−−/++) genotypes fit well with real data measured in previous experiments (Xu et al. 2012a). As might be expected, the presence of a *SUS* locus has a strong effect on 7-alkenes in hypothetical hybrid genotypes. In summary, the significant correlation between *SAD2A*/*SAD5A* allelic expression and the amounts of different alkene classes allowed us to build a relatively accurate and robust model to predict alkene composition based on the expression of *SAD2* and *SAD5*.

### Pollinator attraction as a function of *SAD* genotype

In a previous study, we investigated the influence of alkene composition on pollinator attraction (see fig. 5 in Xu et al. 2012a). Here, we used the alkene composition and pollinator attraction data from that study (Xu et al. 2012a) and modeled the quantitative impact of changes in alkene composition on pollinator attraction by employing a permutation approach (for details, see Methods section). For both pollinators, we found a significant quadratic correlation between pollinator attraction and abundance of alkenes. Our analysis suggests that the attraction (denoted *A*) to *C. cunicularius* and *A. nigroaenea*, respectively, can be modeled as follows: 

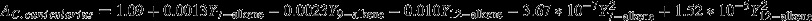
4

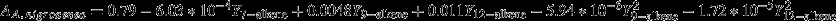
5

**Figure 3:**
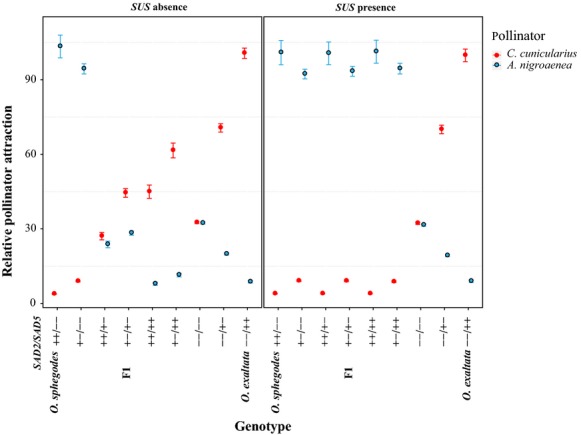
Predicted pollinator attractiveness of each genotype. Red and light blue colors represent attraction to *C. cunicularius* and *A. nigroaenea*, respectively. Left and right panel refer to pollinator attraction in the absence or presence of a *SUS* locus, respectively. The attraction to *C. cunicularius* for each genotype is calculated as a percentage relative to actual *O. exaltata* attraction to *C. cunicularius*. The attraction to *A. nigroaenea* for each genotype is calculated relative to the attraction of *O. sphegodes* scent to *A. nigroaenea*. Mean value and 95% confidence interval are presented.

The first equation (Eq. 4) explained 48.72% of variance of attraction to *C. cunicularius*, and the latter (Eq. 5) explained 30.60% of variance of attraction to *A. nigroaenea*, indicating that floral scent has major effects on pollinator attraction for both *C. cunicularius* and *A. nigroaenea*. These two equations suggest that the attractiveness to either pollinator depends on both absolute amounts and relative ratios of the three alkene groups. We then calculated expected pollination (Fig.3) based on predicted floral alkene composition (from Eq. 4) and the models of pollinator attractiveness (Eq. 4 and 5). Without the presence of the *SUS* locus, *O. sphegodes* and *O. exaltata* genotypes only showed specific attraction to their own pollinator, while the intermediate F_1_ genotype (+−/+−) showed low (<50%) pollinator attraction to both *C. cunicularius* and *A. nigroaenea*, consistent with published data (Xu et al. 2012a). However, with *SUS* present, intermediate genotypes with *SAD2A* (“+”) allele were always highly attractive to *A. nigroaenea*, regardless of *SAD5* genotype. Furthermore, in the absence of *SUS*, the net increase in attractiveness to *C. cunicularius* when gaining a *SAD5* functional allele is 21% on average, whereas the net increase in *A. nigroaenea* attraction on gain of a functional *SAD2* allele is only 3% on average (Fig.3, Tables S1 and S2). These data further suggest that the relative phenotypic effect of *SAD5* is generally greater than that of *SAD2*. In the presence of *SUS*, the relative effect of *SAD2* on pollinator attraction becomes even lower (Table S1). Importantly, there was no genotype that had high (>50%) predicted attractiveness to *both* pollinators at the same time, irrespective of whether *SUS* was assumed to be present or not.

### Simulation of pollination and evolution at the adaptive loci *SAD2* and *SAD5*

**Figure 4:**
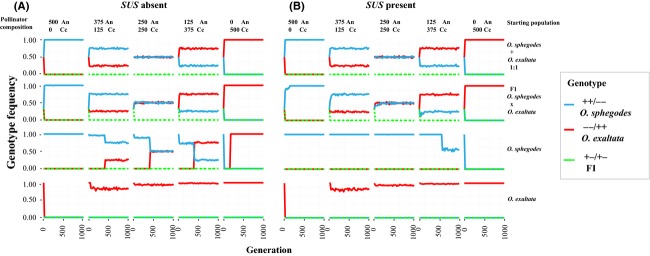
Predicted speciation process between *O. exaltata* and *O. sphegodes*. The *y*-axis in each graph refers to genotype frequency and the *x*-axis to the number of generations. (A) and (B) represent scenarios in the absence or presence of a *SUS* locus. Each column represents different pollinator compositions: “An” refers to *A. nigroaenea*; “Cc” refers to *C. cunicularius*. Each row shows a different starting population (top to bottom): a 1:1 mix of *O. sphegodes*-like and *O. exaltata*-like plants; only *O. sphegodes* × *O. exaltata* F_1_ hybrids; a pure *O. sphegodes*-like population in which one individual carries one *SAD5A* allele; a pure *O. exaltata*-like population in which one individual carries one *SAD2A* allele. Red, green, and light blue color refer to the genotypes of *O. sphegodes*-like, *O. sphegodes* × *O. exaltata* F_1_ hybrid-like, and *O. exaltata*-like plants, respectively.

Using our model of the effect of two *SAD* loci on pollinator attraction, the evolutionary outcome of selection by pollinators was simulated through time, starting with several different initial population compositions. Initial conditions were so chosen as to be informative on the hypothetical speciation process between *O. sphegodes* and *O. exaltata*. Our results (Fig.4) suggest that reproductive isolation between the two orchid species is largely independent of pollinator composition, the presence of a *SUS* locus and the genotype composition of the starting population, given that F_1_ hybrid genotypes are only rarely observed in the different simulated scenarios. This is also consistent with field observations and population genetic data (Xu et al. 2011). Even in a hypothetical population consisting entirely of F_1_ hybrids, pollinators quickly drive populations toward the *SAD* genotypes of the “pure” species (Fig.4, 2nd row).

In the absence of *SUS*, the likelihood of pollinator-mediated speciation between *O. exaltata* and *O. sphegodes* was found to be asymmetric: Our simulated data showed that *O. exaltata* can evolve from *O. sphegodes*, provided that the pollinator of *O. exaltata* (*C. cunicularius*) exists (Fig.4A, rows 3 and 4). However, it seems impossible for *O. sphegodes* to evolve from *O. exaltata* even when only the pollinator of *O. sphegodes* (*A. nigroaenea*) is present. This asymmetric pattern appears to be due to the different effects of *SAD2* and *SAD5* on plant fitness: When a novel functional allele of *SAD5* evolves in *O. sphegodes*, it can increase the relative probability of attracting the new pollinator (*C. cunicularius*) by 19%, but when a novel functional allele of *SAD2* evolves in *O. exaltata*, the associated increase in the probability of attraction of the new pollinator (*A. nigroaenea*) is only 3%. The absence or presence of a *SUS* locus has a strong impact on the possibility of sympatric speciation when starting from “pure” species (Fig.4, comparing rows 3 and 4 of panel A and B); with *SUS,* it was neither possible for *O. sphegodes* to evolve into *O. exaltata*, even when *C. cunicularius* was highly abundant, nor was the opposite evolutionary scenario (from *O. exaltata* to *O. sphegodes*) possible. Furthermore, our model also suggests that the presence of *SUS* increases the frequency of *O. sphegodes* genotypes when *C. cunicularius* and *A. nigroaenea* coexist (Fig.4A and B, 3rd row).

## Discussion

### Effect size and reproductive isolation

In this study, we modeled the effect of two *SAD* loci on pollinator attraction and then used this model to simulate the speciation process under different evolutionary scenarios. Our findings reveal that the relative phenotypic effects of the two adaptive loci *SAD2* and *SAD5* are unequal, and our predictions (Fig.3, Table S2) suggest the effect of *SAD5* to be greater than that of *SAD2*. This may in principle be due to (1) differences in gene expression and hence protein concentration; (2) differences in the enzymatic activities of the proteins; or (3) intrinsic differences in the strength of the pollinators' response to the different alkenes. Gene expression data show higher expression levels of *SAD5* than for *SAD2* (Xu et al. 2012a), consistent with *SAD5*'s higher effect size; this suggests that gene expression differences may contribute to the effect size difference. Our model also predicts that the effect of *SAD2* would further be diminished in the presence of *SUS*. As no genotype (with or without *SUS*) was highly attractive to both pollinators at the same time, this implies that while *SUS* may shift the point in genotypic space where pollination by *Andrena* or *Colletes* becomes likelier, it would not *per se* be expected to strongly affect the strength of the reproductive barrier between the two species. However, *SUS* does affect which pollinator is more likely to visit a potential F_1_ hybrid and thereby the predominant direction of backcrossing on hybridization (with *SUS* present, backcrossing would be toward *O. sphegodes*). In our population simulation, reproductive isolation was also found to be largely independent of pollinator composition and the initial population's genotype composition. Rather, the reproductive barrier between the two species results from differences in floral scent and specific pollinator attraction, which in turn are a consequence of the opposing effects of the two *SAD* loci underlying alkene biosynthesis.

### Evolutionary implications

Knowledge of the genetics of two desaturase loci, *SAD2* and *SAD5*, which effectively control pollinator attraction in two species of *Ophrys* orchids, allowed us to evaluate different evolutionary scenarios of species divergence by modeling. This model predicts the attractiveness of different *SAD2*/*SAD5* genotypes to two pollinator species, representing a proxy of the fitness landscape as a function of two genetic loci (Fig.3). These predictions (Table S1) should be tested experimentally (cf. Nosil and Schluter 2011). Our results show that the potential for pollinator-mediated ecological speciation processes between *O. sphegodes* and *O. exaltata* is likely to be asymmetric due to different effect sizes of alleles at *SAD2* and *SAD5*. This indicates that the effect size distribution of alleles at different loci can contribute to the likelihood of different evolutionary paths within an adaptive landscape, with different consequences with respect to adaptation and speciation. Specifically, the genetic architecture of pollinator attraction predicts that *O. exaltata* is more likely to have evolved from *O. sphegodes* than *vice versa* if a scenario of pollinator-driven sympatric divergence is assumed. This predicted speciation scenario would be consistent with the geographic distributions of those two species, *O. sphegodes* being more widely distributed than *O. exaltata* and therefore a more probable progenitor species a priori (cf. Schlüter et al. 2011a).

Pure species-like genotypes are highly attractive to their respective pollinators as compared to intermediate genotypes. Despite the presence of this apparent “adaptive valley” (Figs.3 and S1), our model suggests that, given sufficient pollinator abundance, intermediate genotypes can be maintained in the population for long enough for novel highly attractive genotypes to evolve; conceptually, this allows the adaptive valley to be crossed. In fact, the “lag time” for *O. exaltata* to evolve from *O. sphegodes* (3rd row of Fig.4A) corresponds to the time taken until the respective genotype is formed in the population; thereafter, its genotype frequency increases rapidly, implying the potential for rapid species divergence. We note that our model considers the de novo origin of an adaptive allele. However, considering that there may be substantial genetic polymorphism within *Ophrys* (Schlüter et al. 2011a; Sedeek et al. in press), adaptive alleles may well have been derived from standing genetic variation. In this case, the initial frequency of the adaptive allele would have been higher (at the time when it became adaptive), and hence, evolution may have proceeded even faster (i.e., less “lag time”).

### Effect and evolution of *SUS*

The possible sympatric speciation scenarios change dramatically in the presence of a *SUS* locus, which effectively prevents evolution from either species to the other. In reality, the frequency of *SUS* in *O. sphegodes* is unknown, so that our simulations reflect the two most extreme outcomes. If *SUS* were fixed or very common in *O. sphegodes*, one might expect that functional *SAD5* alleles can potentially introgress into *O. sphegodes*, as *SUS* renders them effectively neutral; however, (consistent with population genetic data; Xu et al. 2012a) *SAD5* would not be expressed in this species and would instead be expected to accumulate deleterious mutations. It would seem likely that the addition of *SUS* to the genetic architecture of alkene biosynthesis in *O. sphegodes* is more recent than the divergence of the two species, simply because the later addition of a genetic modifier to an already existing genetic program controlling reproductive isolation (where the modifier has no perceptible effect on reproductive isolation *per se*) is the more parsimonious hypothesis. Interestingly, the presence of *SUS* increases the proportion of *O. sphegodes*-like genotypes in situations of decreased *A. nigroaenea* pollinator abundance. Hence, *SUS* may provide a selective advantage to *O. sphegodes* in populations (or years) of low pollinator abundance. It is conceivable that such conditions aided the recruitment of *SUS* into the control of existing alkene biosynthesis in *O. sphegodes*, without a direct effect on reproductive isolation between the two species. Although it appears more plausible to assume that species divergence proceeded without *SUS* (i.e., that this genetic modifier evolved after speciation), this hypothesis remains to be investigated.

## Conclusions

Although previous studies (Schlüter et al. 2011b; Xu et al. 2012a) had revealed that two *SAD* loci have major effects on reproductive isolation and pollinator adaptation in *Ophrys*, how such a genetic architecture influences the evolutionary processes of lineage divergence in this plant group has remained unclear. The results of our modeling approach suggest that the two-locus architecture of reproductive isolation allows for potentially rapid sympatric speciation in *Ophrys* orchids and predicts genetic patterns in line with data from natural orchid populations (e.g., Mant et al. 2005a; Schlüter et al. 2009; Xu et al. 2012a; Sedeek et al. in press[Bibr b20]). More interestingly, our model predicts that the potential for speciation is likely asymmetric in *Ophrys* due the different fitness effects of *SAD2* and *SAD5*. Fluctuating pollinator environments may have favoured the evolution of a suppressor of *SAD5* activity, *SUS*, in *O. sphegodes*. Although this suppressor does not affect reproductive isolation, it may constrain the potential for further evolutionary divergence in sympatry if fixed in a population.

## Supporting Information

**Figure S1.** Scaled probabilities of gene flow among pairs of *SAD2/SAD5* genotypes in the absence or presence of *SUS*. (A, B) Three-dimensional plot for gene flow mediated by the two pollinators shown separately; blue and red surfaces represent gene flow mediated by *A. nigroaenea* (“*An*”) and *C. cunicularius* (“*Cc*”), respectively. Pure species-like and F_1_-like genotypes are indicated. The x- and y-axes indicate different genotypes of *SAD2/SAD5*, and the z-axis indicates the scaled gene flow (with minimum and maximum values scaled to fit the range 0 to 1). Note that the plot is symmetrical with respect to its left–right diagonal. (C-L) Heat maps of scaled probabilities of total gene flow under different pollinator compositions: Only *A. nigroaenea* (“*An*”) present, (C and D), Only *C. cunicularius* (“*Cc*”), (E and F), *An* and *Cc* in 1:1 ratio (G and H), *An* and *Cc* in 3:1 ratio (I and J), and An and Cc in 1:3 ratio (K and L), and in the absence (C, E, G, I, and K) or presence (D, F, H, J, and K) of *SUS*. The color gradient represents the probability of gene flow, from low (blue) to high (red). The x- and y-axes indicate different genotypes of*SAD2/SAD5*. Pure species-like and F_1_-like genotypes are indicated.

**Table S1.** The predicted gene expression of different hypothetic genotypes of *SAD2A* and *SAD5A*, mean alkene composition, mean relative pollinator attraction, and predicted amount of individual alkenes for each hypothetic genotype. “*An*” refers to *A. nigroaenea*; “*Cc*” refers to *C. cunicularius*.

**Table S2.** The net increase in pollinator attraction by gain of one functional allele of *SAD2* or *SAD5*. Values were calculated based on Table S1. Because it is not realistic to assume −−/−− as a starting genotype, we did not calculate values for pollinator attraction changes involving this genotype.
