# Supplementary material for: Modeling the two-locus architecture of divergent pollinator adaptation: how variation in SAD paralogs affects fitness and evolutionary divergence in sexually deceptive orchids
Source: Ecol Evol. 2015 Jan 4;5(2):493–502. doi: 10.1002/ece3.1378 (PMC4314279; doi:10.1002/ece3.1378)
Supplement: Supplementary file 2 [file ece30005-0493-sd2.pdf]

	Genotype		Relative *SAD* expression		Predicted total amount of alkene class (ng)			Predicted relative pollinator attraction	
	SAD2A	***SAD5A***	***SAD2A***	***SAD5A***	**Total Z7**	**Total Z9**	**Total Z12**	***An***	***Cc***
***SUS* Absent**	++	--	0.45	0.01	121.47	598.25	323.33	101.79	4.03
	+-	--	0.23	0.01	117.68	375.03	152.68	95.54	10.26
	++	+-	0.45	0.21	735.78	590.01	76.16	27.90	23.44
	--	--	0.01	0.01	116.41	163.41	40.42	32.70	38.10
	+-	+-	0.23	0.21	725.18	379.29	45.26	29.29	39.58
	++	++	0.45	0.40	2022.11	603.08	35.57	10.45	34.01
	--	+-	0.01	0.21	703.78	167.42	28.24	19.87	73.88
	+-	++	0.23	0.40	1871.42	379.55	30.50	13.74	58.50
	--	++	0.01	0.40	1894.13	165.04	23.90	10.50	100.69
***SUS* Present**	++	--	0.45	0.01	119.99	587.68	320.23	103.38	4.49
	++	+-	0.45	0.01	116.65	588.08	339.25	98.53	4.34
	++	++	0.45	0.01	112.58	586.72	336.60	94.65	4.42
	+-	++	0.23	0.01	118.56	386.35	159.95	82.18	9.07
	+-	--	0.23	0.01	119.38	377.99	159.94	77.38	9.21
	+-	+-	0.23	0.01	120.98	379.53	156.13	74.57	9.44
	--	--	0.01	0.01	120.43	165.08	39.23	20.94	30.14
	--	+-	0.01	0.21	741.38	163.58	28.40	11.89	68.98
	--	++	0.01	0.40	1860.14	164.84	23.94	6.25	100.33

Individual alkenes (ng)

C21-Z7	C23-Z7	C25-Z7	C27-Z7	C21-Z9	C23-Z9	C25-Z9	C27-Z9	C29-Z9	C25-Z12	C27-Z12	C29-Z12	Genotype	
29.63	34.99	48.70	8.15	15.60	38.55	227.64	242.30	74.16	65.97	142.79	114.58	++/--	***SUS* Absent**
28.71	33.90	47.18	7.89	9.78	24.17	142.70	151.89	46.49	31.15	67.42	54.10	+-/--	
179.50	211.94	294.98	49.36	15.38	38.02	224.50	238.96	73.14	15.54	33.63	26.99	++/+-	
28.40	33.53	46.67	7.81	4.26	10.53	62.18	66.18	20.26	8.25	17.85	14.32	--/--	
176.91	208.88	290.73	48.65	9.89	24.44	144.32	153.62	47.02	9.23	19.99	16.04	+-/+-	
493.32	582.46	810.68	135.65	15.72	38.87	229.48	244.26	74.76	7.26	15.71	12.61	++/++	
171.69	202.72	282.15	47.21	4.36	10.79	63.70	67.81	20.75	5.76	12.47	10.01	--/+-	
456.55	539.05	750.27	125.54	9.90	24.46	144.42	153.72	47.05	6.22	13.47	10.81	+-/++	
462.09	545.59	759.37	127.07	4.30	10.64	62.80	66.84	20.46	4.88	10.56	8.47	--/++	
29.27	34.56	48.10	8.05	15.32	37.87	223.61	238.02	72.85	65.34	141.42	113.48	++/--	***SUS* Present**
28.46	33.60	46.77	7.83	15.33	37.90	223.77	238.18	72.90	69.22	149.81	120.22	++/+-	
27.47	32.43	45.14	7.55	15.30	37.81	223.25	237.63	72.73	68.68	148.64	119.28	++/++	
28.93	34.15	47.53	7.95	10.07	24.90	147.01	156.48	47.89	32.63	70.64	56.68	+-/++	
29.12	34.39	47.86	8.01	9.85	24.36	143.83	153.09	46.86	32.63	70.63	56.68	+-/--	
29.51	34.85	48.50	8.12	9.89	24.46	144.41	153.72	47.05	31.86	68.95	55.33	+-/+-	
29.38	34.69	48.28	8.08	4.30	10.64	62.81	66.86	20.46	8.00	17.32	13.90	--/--	
180.87	213.55	297.23	49.74	4.26	10.54	62.24	66.25	20.28	5.80	12.54	10.07	--/+-	
453.80	535.80	745.74	124.79	4.30	10.62	62.72	66.76	20.43	4.88	10.57	8.48	--/++	
