# Supplementary material for: Modeling the two-locus architecture of divergent pollinator adaptation: how variation in SAD paralogs affects fitness and evolutionary divergence in sexually deceptive orchids
Source: Ecol Evol. 2015 Jan 4;5(2):493–502. doi: 10.1002/ece3.1378 (PMC4314279; doi:10.1002/ece3.1378)
Supplement: Supplementary file 3 [file ece30005-0493-sd3.pdf]

	From genotype		To genotype		Net increase in pollinator attraction		Average increase in pollinator attraction	
	SAD2	*SAD5*	*SAD2*	*SAD5*	*A. nigroaenea*	*C. cunicularius*	*A. nigroaenea*	*C. cunicularius*
**Gain of one *SAD2* functional allele**	+-	--	++	--	6.57	6.23	**2.9**	-22.2
	+-	+-	++	+-	-1.39	-16.1		
	--	+-	+-	+-	9.41	-34.3		
	+-	++	++	++	-3.29	-24.5		
	--	++	+-	++	3.24	-42.2		
**Gaining one *SAD5* functional allele**	--	+-	--	++	-9.4	26.8	-36.5	**21.0**
	+-	--	+-	+-	-66.2	29.3		
	+-	+-	+-	++	-15.5	18.9		
	++	--	++	+-	-73.9	19.4		
	++	+-	++	++	-17.4	10.6		
