# Supplementary material for: Modeling the two-locus architecture of divergent pollinator adaptation: how variation in SAD paralogs affects fitness and evolutionary divergence in sexually deceptive orchids
Source: Ecol Evol. 2015 Jan 4;5(2):493–502. doi: 10.1002/ece3.1378 (PMC4314279; doi:10.1002/ece3.1378)
Supplement: Supplementary file 1 [file ece30005-0493-sd1.pdf]

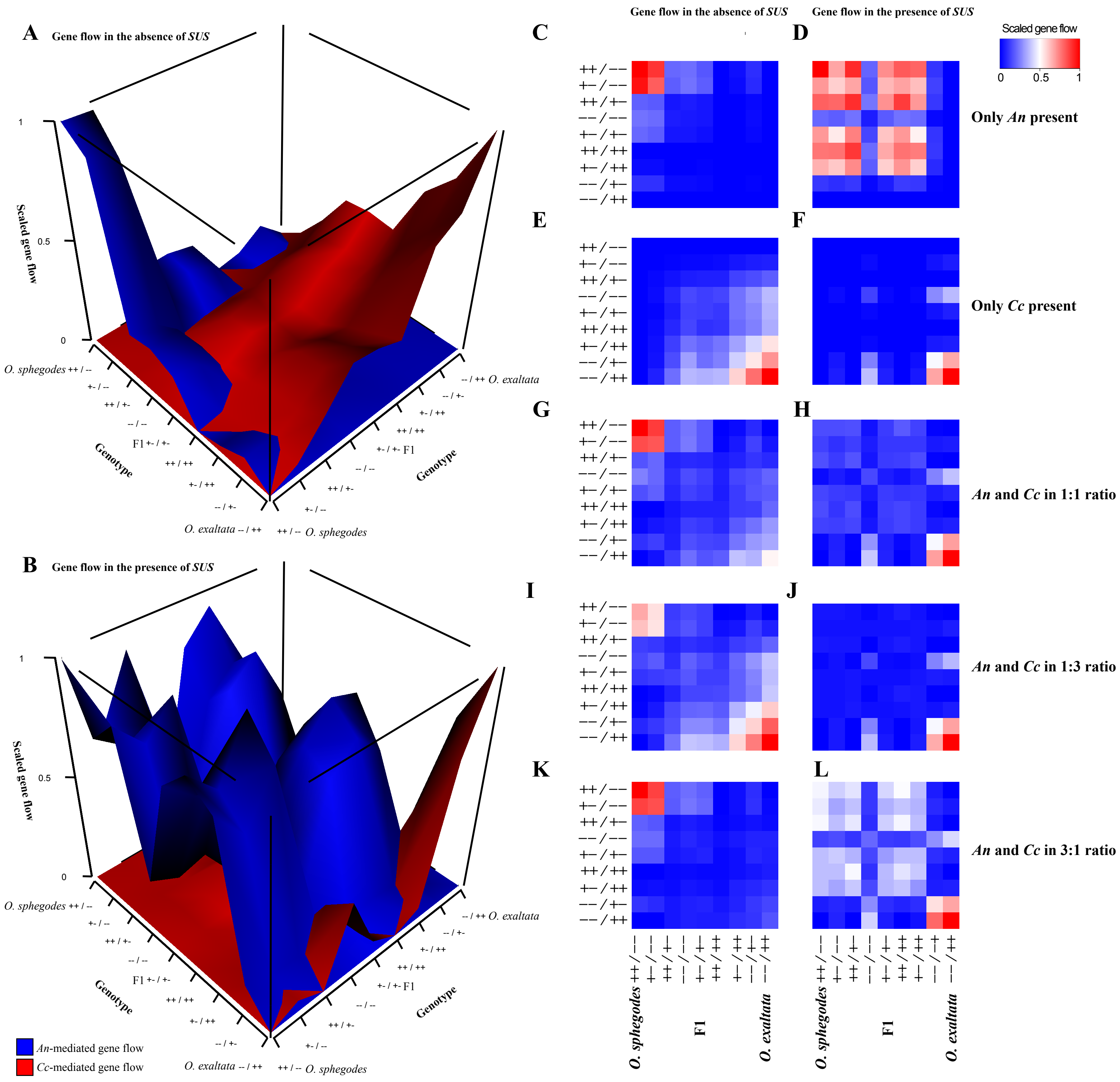

A
Gene flow in the absence of SUS
B
Gene flow in the presence of SUS
Scaled gene flow
1
0.5
0
O. sphegodes ++ / --
+- / --
++ / +-
-- / --
F1 +- / +-
++ / ++
+- / ++
-- / +-
O. exaltata -- / ++
-- / ++ O. exaltata
-- / +-
+- / ++
++ / ++
+- / +- F1
-- / --
++ / +-
+- / --
++ / -- O. sphegodes
Genotype
An-mediated gene flow
Cc-mediated gene flow
Gene flow in the absence of SUS
Gene flow in the presence of SUS
Scaled gene flow
0
0.5
1
C
D
E
F
G
H
I
J
K
L
Only An present
Only Cc present
An and Cc in 1:1 ratio
An and Cc in 1:3 ratio
An and Cc in 3:1 ratio
++ / −−
+− / −−
++ / +−
−− / −−
+− / +−
++ / ++
+− / ++
−− / +−
−− / ++
O. sphegodes
F1
O. exaltata
